# 热消融治疗肺部亚实性结节专家共识（2021年版）

**DOI:** 10.3779/j.issn.1009-3419.2021.101.14

**Published:** 2021-05-20

**Authors:** 欣 叶, 卫君 范, 忠敏 王, 俊杰 王, 徽 王, 俊 王, 春堂 王, 立志 牛, 勇 方, 善智 古, 辉 田, 宝东 刘, 楼 仲, 一平 庄, 嘉昌 池, 锡超 孙, 诺 阳, 志刚 危, 肖 李, 晓光 李, 玉亮 李, 春海 李, 岩 李, 霞 杨, 武威 杨, 坡 杨, 正强 杨, 越勇 肖, 晓明 宋, 开贤 张, 仕林 陈, 炜生 陈, 征宇 林, 殿杰 林, 志强 孟, 晓菁 赵, 凯文 胡, 晨 柳, 澄 柳, 春东 顾, 栋 徐, 勇 黄, 广慧 黄, 忠民 彭, 亮 董, 磊 蒋, 玥 韩, 庆师 曾, 勇 靳, 光焰 雷, 博 翟, 海亮 黎, 杰 潘, 中国医师协会肿瘤消融治疗技术专家组, 中国医师协会介入医师分会肿瘤消融专业委员会, 中国抗癌协会肿瘤消融治疗专业委员会, 中国临床肿瘤学会消融专家委员会

**Affiliations:** 1 250014 济南, 山东第一医科大学第一附属医院(山东省千佛山医院)肿瘤中心, 山东省肺癌研究所 Department of Oncology, The First Affiliated Hospital of Shandong First Medical University & Shandong Provincial Qianfoshan Hospital, Shandong Lung Cancer Institute, Jinan 250014, China; 2 510050 中山, 中山大学肿瘤防治中心微创介入科 Department of Minimally Invasive Interventional Therapy, Sun Yat-sen University Cancer Center, Guangzhou 510050, China; 3 200025 上海, 上海交通大学医学院附属瑞金医院放射介入科 Department of Interventional Radiology, Ruijin Hospital, School of Medicine, Shanghai Jiao Tong University, Shanghai 200025, China; 4 100191 北京, 北京大学第三医院放射治疗科 Department of Radiation Oncology, Peking University Third Hospital, Beijing 100191, China; 5 170412 长春, 吉林省肿瘤医院介入治疗中心 Interventional Center, Jilin Provincial Cancer Hospital, Changchun 170412, China; 6 253022 德州, 德州市第二人民医院胸外科 Department of Thoracic Surgery, Dezhou Second People's Hospital, Dezhou 253022, China; 7 510665 广州, 暨南大学附属复大肿瘤医院肿瘤科 Department of Oncology, Affiliated Fuda Cancer Hospital, Jinan University, Guangzhou 510665, China; 8 310016 杭州, 浙江大学医学院附属邵逸夫医院肿瘤内科 Department of Medical Oncology, Sir Run Run Shaw Hospital, Zhejiang University School of Medicine, Hangzhou 310016, China; 9 410013 长沙, 湖南省肿瘤医院介入科 Department of Interventional Radiology, Hunan Cancer Hospital, Changsha 410013, China; 10 250012 济南, 山东大学齐鲁医院胸外科 Department of Thoracic Surgery, Qilu Hospital of Shandong University, Jinan 250012, China; 11 100053 北京, 首都医科大学宣武医院胸外科 Department of Thoracic Surgery, Xuan Wu Hospital Affiliated to Capital Medical University, Beijing 100053, China; 12 226001 南通, 南通大学附属医院胸外科 Thoracic Surgery Department, Affiliated Hospital of Nantong University, Nantong 226001, China; 13 210009 南京, 江苏省肿瘤医院介入治疗科 Department of Interventional Therapy, Jiangsu Cancer Hospital, Nanjing 210009, China; 14 200127 上海, 上海交通大学医学院附属仁济医院肿瘤介入科 Department of Interventional Oncology, Renji Hospital, School of Medicine, Shanghai Jiaotong University, Shanghai 200127, China; 15 250021 济南, 山东第一医科大学附属省立医院病理科 Department of Pathology, Shandong Provincial Hospital Affiliated to Shandong First Medical University, Jinan 250021, China; 16 530021 南宁, 广西医科大学第一附属医院心胸外科 Department of Cardiothoracic Surgery, The First Affiliated Hospital of Guangxi Medical University, Nanning 530021, China; 17 100021 北京, 中国医学科学院肿瘤医院介入治疗科 Department of Interventional Therapy, Cancer Hospital, Chinese Academy of Medical Sciences and Peking Union Medical College, Beijing 100021, China; 18 100730 北京, 北京医院微创治疗中心 Minimally Invasive Tumor Therapies Center, Beijing Hospital, Beijing 100730, China; 19 250033 济南, 山东大学第二医院介入医学科 Department of Interventional Medicine, The Second Hospital of Shandong University, Jinan 250033, China; 20 250012 济南, 山东大学齐鲁医院放射科 Department of Radiology, Qilu Hospital of Shandong University, Jinan 250012, China; 21 250101 济南, 山东第一医科大学附属省立医院肿瘤中心 Department of Oncology, Shandong Provincial Hospital Afliated to Shandong First Medical University, Jinan 250101, China; 22 100071 北京, 解放军总医院第五医学中心肿瘤科 Department of Oncology, The Fifth Medical Center, Chinese PLA General Hospital, Beijing 100071, China; 23 150001 哈尔滨, 哈尔滨医科大学附属第四医院介入血管外科 Interventionael & Vascular Surgery, The Fourth Hospital of Harbin Medical University, Harbin 150001, China; 24 100036 北京, 中国人民解放军总医院放射诊断科 Department of Radiology, Chinese PLA Gneral Hospital, Beijing 100036, China; 25 250014 济南, 山东第一医科大学第一附属医院胸外科 Department of Thoracic Surgery, The First Affiliated Hospital of Shandong First Medical University & Shandong Provincial Qianfoshan Hospital, Jinan 250014, China; 26 277500 滕州, 山东滕州市中心人民医院肿瘤科 Department of Oncology, Tengzhou Central People's Hospital, Tengzhou 277500, China; 27 210009 南京, 江苏省肿瘤医院胸外科 Department of Thoracic Surgery, Jiangsu Cancer Hospital, Nanjing 210009, China; 28 350011 福州, 福建医科大学附属肿瘤医院胸外科 Department of Thoracic Surgery, Fujian Medical University Cancer Hospital, Fujian 350011, China; 29 350005 福州, 福建医科大学附属第一医院介入科 Department of Intervention, The First Affiliated Hospital of Fujian Medical University, Fujian 350005, China; 30 250021 济南, 山东第一医科大学附属省立医院呼吸与危重症医学科 Department of Respiratory and Critical Care Medicine, Shandong Provincial Hospital Affiliated to Shandong First Medical University, Jinan 250021, China; 31 200032 上海, 复旦大学附属肿瘤医院肿瘤微创治疗中心 Minimally Invasive Therapy Center, Fudan University Shanghai Cancer Center, Shanghai 200032, China; 32 200127 上海, 上海交通大学医学院附属仁济医院胸外科 Department of Thoracic Surgery, Renji Hospital, School of Medicine, Shanghai Jiaotong University, Shanghai 200127, China; 33 100078 北京, 北京中医药大学附属东方医院肿瘤科 Department of Oncology, Dongfang Hospital Affiliated to Beijing University of Chinese Medicine, Beijing 100078, China; 34 100161 北京, 北京肿瘤医院介入治疗科 Department of Interventional Therapy, Beijing Cancer Hospital, Beijing 100161, China; 35 250021 济南, 山东省医学影像研究所CT研究室 Department of Radiology, Shandong Medical Imaging Research Institute, Jinan 250021, China; 36 116011 大连, 大连医科大学附属第一医院胸外科 Department of Thoracic Surgery, The First Affiliated Hospital of Dalian Medical University, Dalian 116011, China; 37 310022 杭州, 中国科学院大学附属肿瘤医院超声医学科 Department of Diagnostic Ultrasound Imaging & Interventional Therapy, The Cancer Hospital of the University of Chinese Academy of Sciences(Zhejiang Cancer Hospital), Hangzhou 310022, China; 38 250117 济南, 山东第一医科大学附属肿瘤医院影像科 Department of Imaging, Affiliated Cancer Hospital of Shandong First Medical University, Jinan 250117, China; 39 250021 济南, 山东第一医科大学附属省立医院胸外科 Department of Thoracic Surgery, Shandong Provincial Hospital Affiliated to Shandong First Medical University, Jinan 250021, China; 40 250014 济南, 山东第一医科大学第一附属医院(千佛山医院)呼吸与危重症医学科 Department of Respiratory and Critical Care Medicine, The First Affiliated Hospital of Shandong First Medical University & Shandong Provincial Qianfoshan Hospital, Jinan 250014, China; 41 214063 无锡, 华东疗养院放射科 Department of Radiology, The Convalescent Hospital of East China, Wuxi 214063, China; 42 250014 济南, 山东第一医科大学第一附属医院(千佛山医院)医学影像科 Department of Medical Imaging, The First Affiliated Hospital of Shandong First Medical University & Shandong Provincial Qianfoshan Hospital, Jinan 250014, China; 43 215004 苏州, 苏州大学附属第二医院介入治疗科 Interventionnal Therapy Department, The Second Affiliated Hospital of Soochow University, Suzhou 215004, China; 44 710061 西安, 陕西省肿瘤医院胸外科 Department of Thoracic Surgery, Shanxi Provincial Cancer Hospital, Xi'an 710061, China; 45 450003 郑州, 河南省肿瘤医院微创介入治疗科 Department of Interventional Radiology, Henan Cancer Hospital, Zhengzhou 450003, China; 46 100730 北京, 中国医学科学院北京协和医院放射科 Department of Radiology, Chinese Academy of Medical Sciences & Peking Union Medical College, Beijing 100730, China

**Keywords:** 肺肿瘤, 筛查, 肺亚实性结节, 肺磨玻璃结节, 热消融, Lung neoplasms, Screening, Pulmonary subsolid nodule, Ground-glass nodule, Thermal ablation

## Abstract

局部热消融技术在肺部结节治疗领域正处在起步与发展阶段，为了肺结节热消融治疗的临床实践和规范发展，由“中国医师协会肿瘤消融治疗技术专家组”“中国医师协会介入医师分会肿瘤消融专业委员会”“中国抗癌协会肿瘤消融治疗专业委员会”“中国临床肿瘤学会消融专家委员会”组织多学科国内有关专家，讨论制定了“热消融治疗肺部亚实性结节专家共识（2021年版）”。主要内容包括：①肺部亚实性结节的临床评估；②热消融治疗肺部亚实性结节技术操作规程、适应证、禁忌证、疗效评价和相关并发症；③存在的问题和未来发展方向。

## Abstract (en)

"The Expert Group on Tumor Ablation Therapy of Chinese Medical Doctor Association, The Tumor Ablation Committee of Chinese College of Interventionalists, The Society of Tumor Ablation Therapy of Chinese Anti-Cancer Association and The Ablation Expert Committee of the Chinese Society of Clinical Oncology" have organized multidisciplinary experts to formulate the consensus for thermal ablation of pulmonary subsolid nodules or ground-glass nodule (GGN). The expert consensus reviews current literatures and provides clinical practices for thermal ablation of GGN. The main contents include: (1) clinical evaluation of GGN, (2) procedures, indications, contraindications, outcomes evaluation and related complications of thermal ablation for GGN and (3) future development directions.

## 1 前言

在世界范围内肺癌发病率虽位居第二，但死亡率仍高居首位^[1]^，因此早期发现、早期诊断、早期治疗是降低肺癌死亡率的重要手段。2011年，美国国家肺癌筛查试验（National Lung Screening Trial, NLST）首次报告了低剂量计算机断层扫描（low-dose computed tomography, LDCT）筛查可以显著降低高危人群肺癌的死亡率，与标准胸部X线检查相比，LDCT筛查可使肺癌病死率降低20%^[2]^。近年来，随着LDCT筛查项目的广泛开展，越来越多的无症状肺结节被发现。肺结节在我国检出率为20%-80%^[3-6]^，LDCT筛查97%以上的肺部结节为良性病变，肺癌的检出率仅为0.7%-2.3%^[2, 4-8]^。过高的检出率可能导致过度诊断、过度治疗、浪费医疗资源及增加受检者焦虑心理^[9-13]^。目前针对肺结节的筛查和处理指南主要有：美国国家综合癌症网络（National Comprehensive Cancer Network, NCCN）、Fleischner学会、美国胸科医师协会（American College of Chest Physicians, ACCP）、亚洲和中国^[14, 15]^等指南，由于指南制定者的专业背景、所属国家或地域不同，至今未达成统一的共识。无论何种指南存在何种差异，最终对于肺结节处理原则是一致的：随访观察和外科手术切除。外科手术的进步，特别是电视辅助胸腔镜手术（video-assisted thoracoscopic surgery, VATS）的普遍应用，使得早期肺癌的治疗疗效、术后并发症和死亡率有了一定的改善^[16-19]^，但是仍有许多问题需要解决。

肺部结节常被认为可能是癌前病变或早期肺癌的征象，而磨玻璃结节（ground-glass nodule，GGN或ground-glass opacity，GGO）样肺癌具有“惰性”发展和极少有远处转移等特点，预后良好，手术切除后5年生存率可达100%^[20-27]^，因此这类肺癌不同于“传统意义”上的早期肺癌，应该是肺癌中的特殊亚型。此类病变过早地应用VATS切除存在一定的问题：①肺结节尤其浸润前病变，过早的手术介入，会导致过早的器官损伤和肺功能损失，术后可能会出现各种并发症，而且早期手术与随访择期手术相比并不能显著改善患者总体生存期；②多发肺结节目前仍无明确的手术方式选择标准，也无剩余结节的后续处理原则；③术前肺结节的诊断是依赖影像学判断，无病理支持，对术前判断有风险的肺结节进行手术切除，术后可能证实为良性病变，使患者经历了不必要的手术和术后并发症^[28-30]^；④随着人口的老龄化，越来越多的早期肺癌患者在75岁以上，这些患者往往无法选择手术治疗。另外，随访也存在问题：每次间隔多久随访？何时终结随访？对于受检者每一次复查都可能带来心理恐慌，甚至严重地影响受检者的生活质量^[31]^，同时也增加了受检者的X射线暴露。为了克服上述问题，需要拓展处理肺结节的新方法。

局部热消融术作为一种精准的微创技术已经应用于早期肺癌的治疗，每年的治疗例数迅速增加^[32-41]^，该技术具有创伤小、疗效明确、安全性高、可重复性强、适应人群广等特点。热消融技术在肺部结节治疗领域正处在起步与发展阶段^[42-49]^，为了推动热消融技术在肺结节治疗中的合理运用，本着安全、有效、规范和可持续发展的原则，由“中国医师协会肿瘤消融治疗技术专家组”“中国医师协会介入医师分会肿瘤消融专业委员会”“中国抗癌协会肿瘤消融治疗专业委员会”“中国临床肿瘤学会消融专家委员会”组织多学科国内有关专家，讨论制定了《热消融治疗肺部亚实性结节专家共识（2021年版）》，以期为热消融治疗肺结节的临床实践和规范发展提供参考。

## 2 肺结节的概念和分类

### 2.1 概念^[50-54]^

由于不同病因造成的肺泡内含气量减少、细胞数量增多、肺泡上皮细胞增生、肺泡间隔增厚和终末气囊内部分充血水肿，这种病理变化在肺部影像学上常表现为：局灶性、边界清楚或模糊、直径（或最大径）≤30 mm、圆形或类圆形、密度增高的阴影。可为单发或多发，不伴有肺不张、肺门及纵隔淋巴结肿大和胸腔积液。

### 2.2 分类

#### 2.2.1 按病变性质^[55]^

① 良性：良性肿瘤、各种感染性疾病、风湿类疾病、先天性疾病、肺出血等；②恶性：肺癌（浸润前病变、浸润性癌）、淋巴瘤、肉瘤、肺转移瘤等。

#### 2.2.2 按密度

可分为实性和亚实性肺结节。①实性肺结节（solid nodule）：CT肺窗观察，肺内圆形或类圆形的密度增高病变，可掩盖其内走行的血管和支气管，纵隔窗图像显示为软组织密度；②亚实性肺结节（subsolid nodule）^[28, 53, 56, 57]^：CT肺窗观察，肺内圆形或类圆形的高密度病变，不掩盖其内走行的血管和支气管影，纵隔窗图像不显示，类似为磨玻璃样，因此称为GGN或GGO。亚实性肺结节又分为：纯GGN（pure GGN, pGGN）和磨玻璃密度中带有实性密度成分的混合性GGN（mixed GGN, mGGN），后者也称部分实性结节（partial solid nodule, PSN）。在亚实性肺结节中如果是潜在恶性或恶性其病理类型为肺腺癌相关的组织亚型^[53, 58-62]^，可涉及从肺泡上皮不典型腺瘤样增生（atypical adenomatous hyperplasia, AAH）到原位腺癌（adenocarcinoma *in situ*, AIS），到微浸润腺癌（microinvasive lung adenocarcinoma, MIA），再到浸润性腺癌（invasive adenocarcinoma, IAC）等多个腺癌演进阶段。本共识主要述及亚实性肺结节即GGN。

#### 2.2.3 按大小^[14, 57]^

① 微小结节：直径 < 5 mm（体积 < 100 mm^3^）；②小结节：直径5 mm-10 mm（体积100 mm^3^-400 mm^3^）；③结节：直径11 mm-30 mm（体积 > 400 mm^3^）。

#### 2.2.4 按数量^[63-65]^

① 单发：单个病灶；②多发：2个及以上的病灶。

#### 2.2.5 按危险因素^[2, 3, 14, 15]^

① 高危因素结节：年龄≥50岁且具有下列一种危险因素者：①吸烟≥20包年（或400年支），或曾经吸烟≥20包年（或400年支），戒烟时间 < 15年；②有环境或高危职业暴露史（如石棉、铍、铀、氡等接触者）；③合并慢性阻塞性肺疾病、弥漫性肺纤维化或既往有肺结核病史者；④既往罹患恶性肿瘤或有肺癌家族史者；⑤低危因素结节：不具备上述危险因素者。近年来发现了许多年龄40岁-50岁、不吸烟、无环境或高危职业暴露史、不合并慢性阻塞性肺疾病、无弥漫性肺纤维化的女性表现为GGN样的肺癌患者^[3-5, 66-68]^。其原因并不清楚，可能是与雌激素-受体介导的信号通路促进了女性肺腺癌的发生有关^[69]^，也不排除部分中国非吸烟女性有长期被动吸烟史（香烟、烹饪烟雾）这一潜在影响因素^[70]^。

按照结节大小和密度分类是最常用的分类方法，也是本共识的主要分类方法。

## 3 CT影像学评估

### 3.1 CT检查参数及测量

#### 3.1.1 扫描参数^[14, 71-74]^

CT是诊断肺部GGN的首选方法，强调薄层低剂量高分辨率CT扫描、靶扫描或靶重建，不需要注射对比剂。CT扫描探测器≥16排，扫描准直层厚：①建议1 mm薄层重建。如扫描层厚 > 1 mm，重建间隔选择准直层厚的50%-80%。重建图像矩阵512×512（最好选择1, 024×1, 024）；②总辐射暴露剂量为1.0 mSv，120 kV，mAs≤40；③窗宽窗位：推荐肺窗窗位为-700 HU--600 HU，窗宽为1, 500 HU-1, 600 HU。纵隔窗窗位为30 HU-70 HU，窗宽为350 HU-400 HU；④扫描范围：深吸气末扫描，从肺尖到肋膈角，扫描采样时间≤10 s。CT筛选推荐低剂量扫描，发现GGN后，推荐应用常规剂量进行靶扫描，以更精准地评估肺部GGN的结构。

#### 3.1.2 测量及观察^[75, 76]^

有关肺部GGN的大小、体积、密度、形态、边缘、内部结构、增长等情况的测量及观察，是制定肺部GGN处理策略的最关键技术指标。本共识基于实用性、可操作性、可重复性和有一定循证医学证据等原则达成如下共识：①测量单位：所有尺寸参数应精确至mm或mm^3^；②结节大小：肺窗下横截面最大长径；③结节体积：根据对结节的分割结果和结节所包含体素的数量计算结节体积；④结节实性成分测量：采用肺窗和纵隔窗相结合的方法，以肺窗测量为主（测量最大横截面长径）^[77-79]^；⑤实性成分与肿瘤比率（consolidation tumor ratio, CTR）计算：肺窗下结节的横截面实性成分最大长径与结节最大长径之比；⑥密度、形态、边缘、内部结构：采用肺窗和纵隔窗相结合的方法，同时可以在不同轴位上观察，必要时三维重建；⑦体积倍增时间（volume-doubling time, VDT）：VDT是判断GGN良恶性的重要参数之一^[80-82]^，一般良性病变≥800 d，浸润前或微浸润病变为400 d-600 d，浸润性病变 < 400 d，传统意义上肺癌为100 d-300 d；⑧人工智能（artificial intelligence, AI）技术^[83, 84]^：鉴于目前AI软件之间差异较大，如果在同一个医疗机构、用同型号CT扫描、同一种处理软件包，并且持续在该医疗机构长期随访复查，AI结果有较大的参考价值。

### 3.2 CT影像学评估

目前临床影像学上尚无评判GGN良恶性的统一标准，临床上常根据肺部GGN的影像学特征如大小、形态、边缘、瘤-肺界面、内部结构特征、位置及随访的动态变化来预测，其中以结节大小、内部结构特征（特别是实性成分）和随访的动态变化最为重要^[79, 85, 86]^。

#### 3.2.1 GGN大小

① 微小结节：直径 < 5 mm（体积 < 100 mm^3^），95%-99%为良性病变；②小结节：直径5 mm-10 mm（体积100 mm^3^-400 mm^3^），80%-85%为良性病变或浸润前病变；③结节：直径11 mm-30 mm（体积 > 400 mm^3^），如果经过3个月-4个月观察随访，不消失或不缩小并持续存在的GGN，60%-80%为浸润前病变或浸润性病变^[63, 82, 87-91]^。

#### 3.2.2 形态

大多数恶性GGN的形态为圆形或类圆形，靠近叶间裂或大血管旁的恶性GGN出现不规则形态的比例较高。

#### 3.2.3 边缘^[92-94]^

如果GGN呈分叶状（以浅分叶多见），或有毛刺征、胸膜凹陷征及血管集束征等征象常提示恶性的可能。炎性GGN边缘多模糊甚至有渗出样改变，良性非炎性肺结节边缘多整齐光滑。如果GGN边缘有尖角或纤维条索或周围出现纤维条索、胸膜增厚等征象则常提示结节为良性。

#### 3.2.4 内部结构特征

① CT值^[95-101]^：目前认为当CT值> -450 HU在病理上通常是浸润性病变，但是由于GGN的面积较小，测量数值重复性较差，临床应用价值尚不能肯定；②CTR：mGGN中的实性成分是决定预后的主要因素，如果mGGN≥15 mm、CTR≥25%时在病理上通常是浸润性病变。CTR的增加或GGN整体增加或两者同步增加都预示着与浸润性肺癌的风险高度相关^[79, 102-104]^；③结节其他征象：空泡征、支气管充气征、血管在结节内扭曲或扩张等征象均提示GGN倾向于浸润性腺癌^[105-107]^。

### 3.3 随访的动态变化

发现GGN后随访是必须采取的措施。40%-50%的GGN在随访3个月-4个月后消失，这些消失的GGN多考虑是炎症^[55, 108-110]^。将随访3个月-4个月后消失的称为暂时性GGN，反之称为持续性GGN。持续性GGN有潜在恶性的可能，这种潜在恶性的GGN要经过较长时间的发展才可能演变成为恶性。因此发现GGN要采用“观察-等待”（watchful-waiting）的方法进行一定时间的随访，观察其动态变化，以便确定GGN的性质^[111-114]^。pGGN和mGGN随访策略有所不同，但是在随访过程中出现如下情况多考虑为恶性：①病灶最大径或体积增大，VDT符合肿瘤生长规律；②病灶稳定或增大，并出现实性成分；③病灶稳定，但实性成分增加；④出现其他恶性征象：如分叶征、毛刺征、胸膜凹陷征、支气管充气征、血管集束征及血管在结节内扭曲或扩张等征象。随访过程中GGN发生增大和实性成分变化是核心指标，可参考图 1进行随访和处理。

**图 1:**
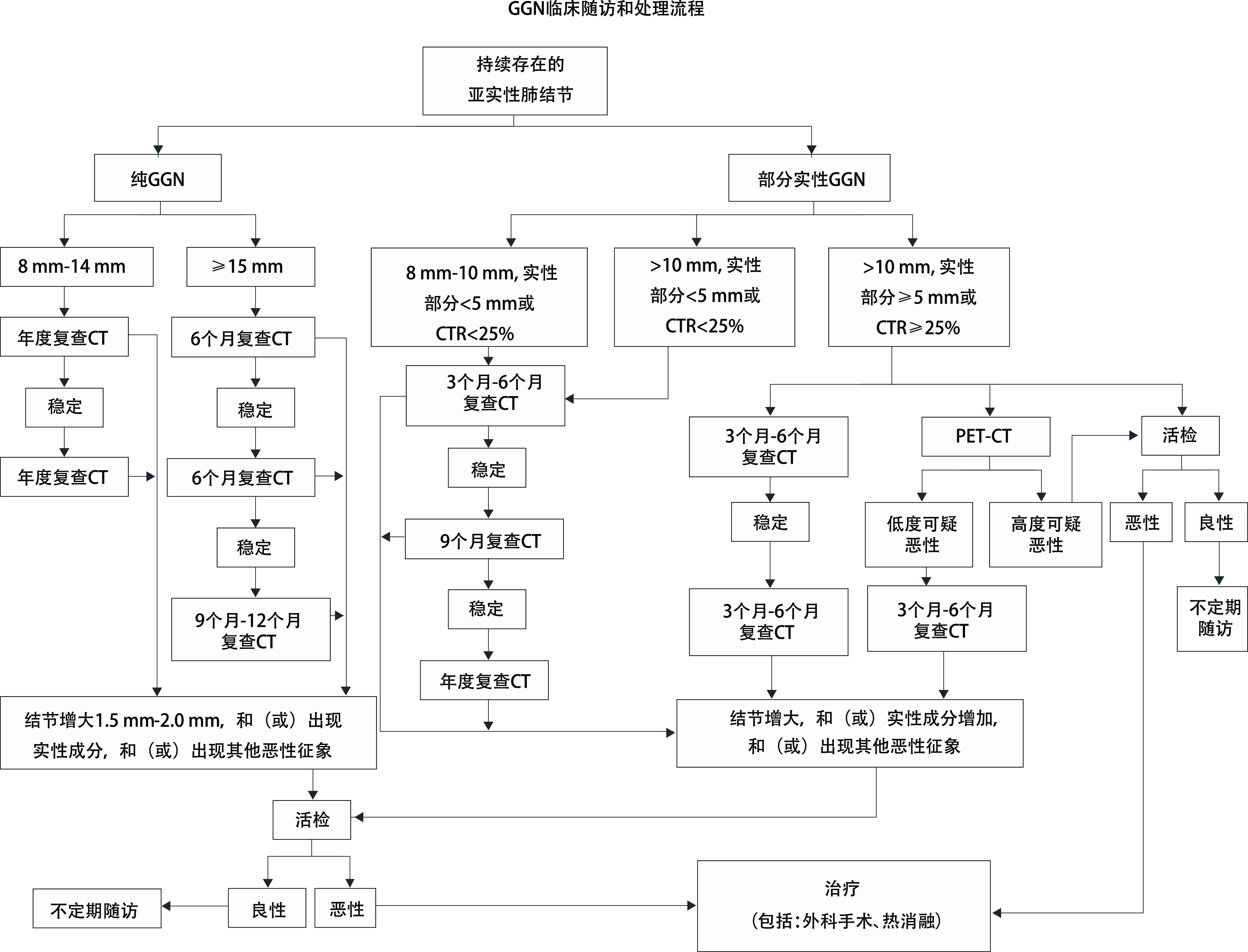
GGN临床随访和处理流程 Clinical follow-up and treatment of GGN. CT: computed tomography; GGN: ground-glass nodule; PET: positron emission computed tomography; CTR: consolidation tumor ratio.

### 3.4 多发GGN

多发GGN是指肺内存在两个或以上最大径均≤30mm的GGN，占肺部GGN的40%-50%。多发GGN有同侧肺同肺叶内多发、同侧肺不同肺叶内多发、双侧肺不同肺叶内单发或多发等多种类型。多发GGN主要病理类型为肺腺癌相关的组织亚型，涵盖了从AAH到AIS再到MIA最后到IAC等多个腺癌发展状态，甚至还可出现良恶性共存的情况^[58, 63, 115-119]^。由于多发GGN的多样性和复杂性，其处理手段仍未达成统一的共识^[65, 120-122]^。目前研究认为多发GGN每个病灶是“独立的个体”而非转移病灶^[63-65, 123-125]^，对于多发GGN的处理应遵循先“主”后“次”的原则，即先处理主病灶，再处理次病灶^[31]^。主病灶依据最大病灶来确定，但有时也用高度怀疑恶性的病灶来确定。多发GGN的预后取决于主病灶大小和实性成分，次要或残留病灶是否生长或是否有新发病灶一般不影响预后^[65, 124, 126-128]^。

## 4 正电子发射型计算机断层显像（positron emission computed tomography, PET）-CT

功能显像是进一步协助区分GGN良恶性的重要方法之一，但是PET-CT对GGN病变的诊断价值有限^[129-134]^。①pGGN：无论大小均不推荐PET-CT检查；②长径≤10 mm、实性成分 < 5 mm的mGGN，不推荐PET-CT；③长径11 mm-15 mm、实性成分≥5 mm的mGGN，推荐PET-CT进行定性，但存在较高的假阴性；④长径 > 15 mm、实性成分≥5 mm的mGGN，定性困难时推荐PET-CT，阳性率较高；⑤伴有肺内其他实性结节，或者有肺外恶性肿瘤病史的GGN患者，推荐行PET-CT检查；⑥PET-CT还可为选择穿刺活检部位提供重要依据。

## 5 活检

病理穿刺活检是明确GGN性质和决定治疗方式重要手段，经胸壁肺穿刺活检技术（percutaneous thoracic needle biopsy, PTNB）和经支气管镜活检是最常用的两种非手术活检技术。

### 5.1 PTNB

#### 5.1.1 PTNB适应证^[135-144]^

在CT引导下进行，参照图 1。pGGN：①最大径 < 8 mm不主张活检；②最大径8 mm-14 mm，在随访过程中增大或出现实性成分；③最大径≥15 mm或在随访过程中增大或出现实性成分。mGGN：①最大径 < 8 mm，实性部分 < 5 mm或CTR < 25%不主张活检；②最大径8 mm-10 mm，实性部分 < 5 mm或CTR < 25%，在随访过程中增大或实性成分增多；③最大径 > 10 mm（实性部分 < 5 mm或CTR < 25%），在随访过程中增大或实性成分增多；④最大径 > 10 mm（实性部分≥5 mm或CTR ≥25%），在随访过程中增大或实性成分增多；⑤最大径 > 10 mm（实性部分≥5 mm或CTR≥25%），PET-CT检查高度怀疑恶性。

#### 5.1.2 PTNB禁忌证

PTNB除不可纠正的凝血功能障碍外绝对禁忌证很少^[135, 137]^。相对禁忌证：①严重恶病质、严重心肺功能不全；②严重慢性阻塞性肺疾病、肺气肿、肺纤维化；③严重肺动脉高压；④机械通气（呼吸机）患者；⑤发作期精神病患者。

#### 5.1.3 PTNB诊断准确率^[145-148]^

① 直径≤8 mm的肺结节准确率为70%-75%；②直径9 mm-10 mm的肺结节诊断准确率为80%-85%；③直径11 mm-20 mm的肺结节诊断准确率为85%-95%；④PTNB与手术后腺癌各亚型符合率为55%-60%^[144, 149]^。

#### 5.1.4 PTNB辅助技术

① 消融后活检^[150-156]^：PTNB术中出现肺实质出血是影响诊断准确率的主要因素，微波消融或射频消融可以凝固肺内2 mm左右的小血管，消融后再取活检能减少肺实质出血，提高活检的阳性率。具体技术操作参考有关文献^[150-154]^；②3D模板技术^[157, 158]^：PTNB尤其是针对下叶GGN的活检存在一定难度，应用3D打印共面坐标模板联合固定针技术可以使GGN相对固定而减小呼吸运动对活检的影响，提高活检的阳性率。

### 5.2 气管镜检查

传统技术包括气管镜直视下刷检、活检或透视下经支气管镜肺活检及支气管肺泡灌洗获取细胞学和组织学对GGN的诊断价值不大。其他新发展的技术包括支气管内超声引导下肺活检术（endobronchial ultrasound-guided transbronchial needle aspiration, EBUS-TBLB）、虚拟导航气管镜（virtual bronchoscopic navigation, VBN）、电磁导航气管镜（electromagnetic navigation bronchoscopy, ENB）。采用可活检的超细气管镜，在其引导下超细气管镜可进入到第5-8级支气管用于GGN的活检^[159-161]^。

## 6 局部热消融技术及影像引导方式

### 6.1 局部热消融技术

肿瘤热消融是针对某一脏器中特定的一个或多个肿瘤病灶，利用热产生的生物学效应直接导致病灶组织中的肿瘤细胞发生不可逆损伤或凝固性坏死的一种治疗技术。目前用于GGN治疗的主要包括射频消融（radiofrequency ablation, RFA）、微波消融（microwave ablation, MWA）和冷冻消融（cryoablation）。

#### 6.1.1 RFA

RFA是目前治疗实体瘤最广泛的消融技术，其原理是将射频电极穿刺入肿瘤组织中，在375 kHz-500 kHz的高频交变电流作用下，肿瘤组织内的离子相互磨擦、碰撞而产生热生物学效应，局部温度可达60 ℃-120 ℃，当组织被加热至60 ℃以上时，可引起细胞凝固性坏死。RFA消融体积取决于局部射频消融产生的热量传导与循环血液及细胞外液间的热对流，易受组织特性的影响^[32, 33, 162]^。

#### 6.1.2 MWA

MWA一般采用915 MHz或2, 450 MHz两种频率。在微波电磁场的作用下，肿瘤组织内的水分子、蛋白质分子等极性分子产生极高速振动，造成分子之间的相互碰撞、相互摩擦，在短时间内产生高达60 ℃-150 ℃的高温，从而导致细胞凝固性坏死。由于辐射器将微波能集中在一定范围内，故而能有效地辐射到所需靶区，微波热辐射在肺内有更高的对流性和更低的热沉降效应^[32, 33, 162]^。

#### 6.1.3 冷冻消融

常用的冷冻消融技术包括氩-氦冷冻消融和液氮冷冻系统。氩-氦冷冻消是通过焦耳-汤姆逊（Joule-Thomson）效应，高压氩气可以使靶组织冷却至-140 ℃，氦气可使靶组织从-140 ℃迅速上升至20 ℃-40 ℃。液氮冷冻消融可以使靶组织冷却至-196 ℃，用无水乙醇升温至80 ℃，通过这种温度梯度的变化可以导致^[32, 33, 162]^：①靶组织蛋白质变性；②细胞内外渗透压改变和“结冰”效应造成细胞裂解；③微血管栓塞引起组织缺血坏死等。用CT或磁共振成像（magnetic resonance imaging, MRI）观察到的“冰球”可以直接将消融区域与肿瘤边界进行区分，可以测定冷冻损伤的边界。

上述3种技术目前在治疗GGN方面均有应用^[43-47, 163, 164]^，但是由于肺脏和GGN具有相对特殊的组织结构，MWA热辐射在肺内有更高的对流性和更低的热沉降效应，因此MWA对于治疗GGN具有一定的优势^[41, 46, 47]^。

### 6.2 影像引导技术

由于GGN影像学上的特殊性，CT是GGN消融治疗最常用的影像引导技术。穿刺时建议CT扫描层厚2.0 mm-2.5 mm，在肺窗下或合适的窗宽和窗位操作。

## 7 热消融治疗肺GGN的适应证和禁忌证

肺癌患者的预后主要取决于是否存在肺门、纵隔淋巴结转移和远处转移。GGN样腺癌是肺腺癌的特殊亚型，主要是局部缓慢生长，而且有着不同的发展阶段，在AAH、AIS和MIA阶段几乎不出现淋巴结和远处转移。即便是IAC阶段，如果最大径≤30 mm、CTR≤50%也极少出现淋巴结和远处转移^[20-27]^。因此，热消融作为局部治疗的有效方法之一，完全可以通过热生物学效应治疗GGN，并且能够实现治愈性消融。治愈性消融是指通过热消融治疗，使局部肿瘤组织完全坏死，有可能达到治愈效果^[32, 165, 166]^。

### 7.1 适应证

#### 7.1.1 周围型GGN患者

① 因心肺功能差或高龄不能耐受手术切除；②拒绝行手术切除；③外科切除后又新出现的病灶或遗留病灶，患者无法耐受再次手术或拒绝再次手术；④多发GGN（先消融主病灶，其他病灶根据发展情况考虑再次消融）；⑤各种原因导致的重度胸膜黏连或胸膜腔闭锁；⑥单肺（各种原因导致一侧肺缺如）；⑦重度焦虑，经心理或药物治疗无法缓解。上述患者需经活检病理证实为AAH、AIS和MIA，对于周围型GGN样IAC患者需排除远处转移。

#### 7.1.2 临床上常遇到几种既拒绝活检又拒绝手术的特殊患者

① 有高危因素，影像学上有恶性征象（如病灶最大径≥15 mm、毛刺征、分叶征、胸膜凹陷、空泡征、血管集束征、动态观察GGN增大、出现实性成分或实性成分增加等）；②虽然没有高危因素，但是影像学上有恶性征象（如病灶最大径 > 15 mm、毛刺征、分叶征、胸膜凹陷、空泡征、血管集束征、动态观察GGN增大、出现实性成分或实性成分增加等）；③发现GGN后极度紧张和焦虑，经心理或药物治疗无法缓解^[10, 31, 167-169]^。对于上述3种患者建议：首先多学科会诊（multidisciplinary team, MDT）共同讨论做出初步诊疗意见，在MDT的基础上与患者共同决策（shared decision making, SDM）制定最终诊疗意见^[170-173]^。如果SDM意见是：“可不取病理直接消融或消融与活检同步进行”，那么医疗人员和患者及其家属（或监护人等）最终可按照SDM意见执行。SDM^[174, 175]^是指在进行医疗和护理决策时，医务人员首先充分告知患者及其家属（或监护人等）各种诊疗措施的利弊、潜在的益处和风险，患者及其家属（或监护人等）通过权衡这些利弊，与医务人员充分沟通，最后共同做出决策。SDM是循证医学的重要内容之一，并且作为一种新型医疗模式，越来越受到关注。

### 7.2 禁忌证

#### 7.2.1 绝对禁忌证

① 血小板 < 50×10^9^/L；②有严重出血倾向、短期内不能纠正的凝血功能障碍（凝血酶原时间 > 18 s，凝血酶原活动度 < 40%）；③严重的肺纤维化和肺动脉高压；④抗凝治疗和（或）抗血小板药物在消融前停用未超过5 d-7 d，贝伐珠单抗末次使用间隔未超过1个月。

#### 7.2.2 相对禁忌证

① 胸腔积液控制不佳者；②肝、肾、心、肺、脑功能严重不全者；③严重贫血、脱水及营养代谢严重紊乱，无法在短期内纠正或改善者；④严重全身感染、高热（> 38.5 ℃）者；⑤美国东部肿瘤协作组（Eastern Cooperative Oncology Group, ECOG）评分 > 3分者；⑥发作期精神病患者；⑦合并其他肿瘤并有广泛转移者，预期生存期 < 6个月；⑧植入心脏起搏器的患者使用RFA时要在充分评估患者心功能的情况下，可考虑RFA手术期间停止起搏器，手术后恢复起搏器。

## 8 术前准备

### 8.1 患者的评估及影像学检查

要通过认真复习病史、体格检查及近期的影像资料来评估患者的热消融适应证。适应证的选择建议MDT多学科（胸外科、肿瘤科、呼吸科、放射治疗科、介入医学科、影像科、病理科等）共同讨论做出决定，必要时进行SDM。胸部薄层CT（层厚≤1 mm，1个月内，可以不用强化）为消融治疗前评估的关键影像学检查，通过CT观察GGN的大小、形态、内部结构、位置及其与邻近重要脏器、血管、气管或支气管的关系。完善相关检查，如高度怀疑是肺部GGN处于Ⅰa阶段可以在消融前行PET-CT检查或全身其他检查排除或发现远处转移。

### 8.2 各项实验室检查

实验室检查项目应包括：血常规、大小便常规、凝血功能、肝肾功能、血糖、肿瘤标志物、血型、血清传染病学等检查，心电图、肺功能、心脏彩超（高龄患者可选）等。

### 8.3 病理检查

在GGN消融前可行PTNB或行各种纤维支气管镜活检以明确诊断。

### 8.4 药品及监护设备准备

术前应准备麻醉、镇痛、镇咳、止血、扩血管、降压等药物，抢救药品及设备。

### 8.5 患者准备

① 患者及（或）家属（被委托人）签署知情同意书，要充分告知患者及其家属（或监护人等）各种诊治方法潜在的获益和风险，积极鼓励参与SDM；②局部麻醉前4 h禁食，全身麻醉前12 h禁食、前4 h禁水；③手术区必要时备皮、建立静脉通道、术前口服镇咳剂和必要的镇静药物；④患者术前教育，主要是呼吸训练。

## 9 麻醉与消毒

根据患者的状况，可以采用全身麻醉或局部麻醉进行消融手术^[32]^。穿刺点处用1%-2%利多卡因局部浸润麻醉，直至胸膜。对于术中不能配合、预计手术时间长、肿瘤贴近壁层胸膜可能引起较严重疼痛的患者，建议全身麻醉。严格执行无菌操作技术规范。

## 10 消融操作

选择合适的消融技术后，CT是最常用和最准确的影像引导方式，操作过程是将热消融针在CT引导下通过皮肤直接精准地穿刺入靶组织中进行消融。消融的操作流程见图 2。

**图 2:**
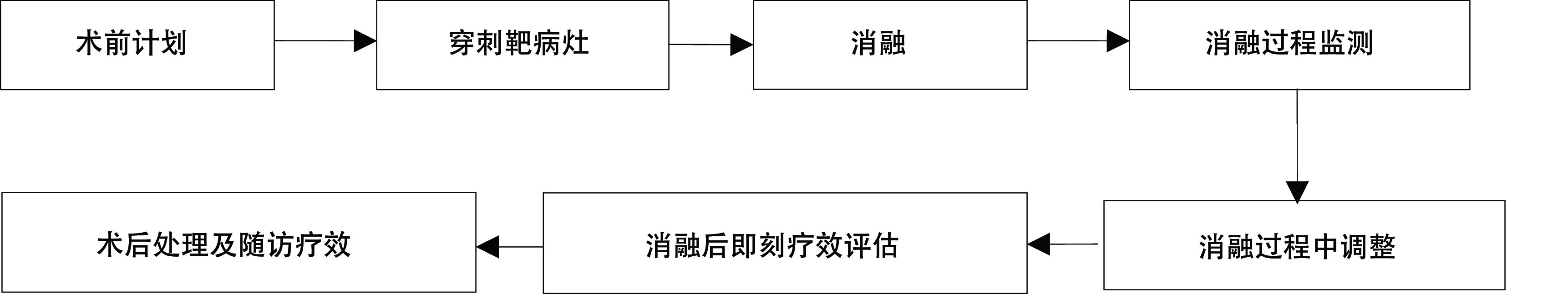
消融的操作流程 Chart ablation procedure

### 10.1 术前治疗计划

术前治疗计划是保证消融是否成功的关键环节。主要包括：①确定病变区域（gross tumor region, GTR）：指影像学能界定的病变区域，即确定病灶的位置、大小、形态、与邻近器官的关系，初步确定GTR；②选择合适体位及穿刺点的体表定位；③穿刺路径：指从穿刺点到达病灶的穿刺通道，此距离称为“靶皮距”；④初步制定消融参数。

### 10.2 穿刺临床靶区

麻醉后用消融针按照术前计划的GTR，从体表定位点沿着穿刺路径逐层穿刺，分步进针，穿刺深度为术前计划的“靶皮距”，然后CT扫描观察（可通过三维重建影像确认）消融针是否到达预定的消融靶区。

### 10.3 消融靶组织

根据肿瘤的大小和部位可采用多种模式进行靶组织消融治疗：①单次单点完成消融；②单次多点完成消融；③多针单次多点；④对于多发病灶多点单次（每次消融≤3个病灶）或多次多点（双肺病灶间隔15 d左右）完成消融。所使用的消融参数（温度、功率、时间、循环等）根据不同的设备进行不同选择。

### 10.4 消融过程中监测

在消融过程中要监测消融针是否脱靶、是否需要调整消融针、是否达到了预定消融范围、是否有术中并发症（如出血、气胸）。热消融过程中，由于热消融对GGN周围肺组织的损伤，在GGN周围可出现不透明高密度区，称为消融后GGO，当GTR周围的GGO大于消融前GTR边界5 mm-10 mm时，消融针可以拔出。此时的靶组织定义为：消融后靶区（post-ablation target zone, PTZ）。消融过程需要监测心率、血压和血氧饱和度，同时要观察患者的呼吸、疼痛、咳嗽、咯血等情况，必要时应对症处理。

### 10.5 即刻疗效评价

① 初步评价操作技术的成功情况；②观察消融边界建议：如果要达到完全消融，PTZ周围的消融后GGO至少要大于消融前GTR边界5 mm；③观察是否有并发症的发生。

### 10.6 术后处理

术后建议监测生命体征，24 h-48 h后CT扫描：①观察消融范围；②观察是否有并发症的发生（如无症状性气胸或胸腔积液）。

## 11 随访及疗效评估

### 11.1 随访

术后1个月复查胸部CT，3个月后再复查胸部CT，主要观察局部病灶是否完全消融以及并发症等。以后每6个月复查胸部CT，主要观察局部病灶是否复发、是否逐渐形成疤痕、肺内是否有新发病灶等。两年后改为年度复查CT。

### 11.2 术后影像学特征及疗效评估

#### 11.2.1 CT疗效评估

##### 11.2.1.1 影像学表现^[32, 176]^

消融后由于消融区周围的出血、水肿、渗出、炎性细胞的浸润，PTZ显著大于原病灶的GTR，而这种影像学特征将持续3个月-4个月，因此传统的实体瘤疗效评价标准（Response Evaluation Criteria in Solid Tumors, RECIST）不适合用于消融后局部疗效的评价，特别是GGN。消融后CT扫描显示的变化规律为：消融后1个月-3个月内病灶增大，3个月后病灶保持稳定或逐渐缩小。①早期改变（1周内）：可分为3层：a.内层：病灶内可出现实性或低密度泡影样改变；b.中间层：围绕着消融病灶周边形成的消融后GGO，一般认为GGO应超出肺结节周边边缘至少5 mm可达到肺GGN完全消融；c.外层：在GGO外有一层密度稍高于GGO的反应带。这种典型的影像学改变称为：“帽徽（cockade）”征象或“煎蛋（friedegg）”征（此征象在消融后24 h-48 h更加明显）；②中期（1周-3个月内）：消融区可持续增大，消融后GGO消失，其周边可能出现环绕清晰锐利的强化环，称为“蛋壳”（egg shell）征象；③后期（3个月后）：与基线（一般以消融后4周-6周时的CT表现为基线）比PTZ在消融治疗3个月后病灶保持稳定，随后的CT随访过程中病灶区域有几种不同的演变模式：如消失、缩小纤维化、空洞、结节、肺不张、增大（可能增生纤维化）等。冷冻消融术后的影像学变化特征与射频和微波消融相比有一定的差异，但可以参考上述变化过程。

##### 11.2.1.2 局部疗效评估^[32, 162, 176]^

以消融后4周-6周时的病灶为基线判断疗效。①完全消融（出现下列表现任何一项）：病灶消失、完全形成空洞、病灶纤维化（可为疤痕）、实性结节缩小或无变化或增大（但CT扫描无造影剂异常强化征象）、肺不张（肺不张内的病灶CT扫描无造影剂异常强化征象）；②不完全消融（出现下列表现任何一项）：a.在形成空洞形成边缘、在病灶纤维化边缘仍有典型的GGN影像学表现；b.病灶部分纤维化仍存有部分实性成分，且实性部分CT扫描强化和（或）PET-CT肿瘤有代谢活性；c.实性结节，大小无变化或增大，且伴CT扫描造影剂有异常强化征象和（或）PET-CT结节有异常代谢活性。

### 11.3 临床疗效评估

在判断局部疗效的基础上，定期随访评价临床疗效^[32, 162]^。①技术成功和安全性评价至少随访6个月；②初步临床疗效评价至少随访1年；③中期临床疗效评价至少随访3年-5年；④长期临床疗效评价至少随访6年-10年。

## 12 并发症及处理

肺结节消融术是一种相对安全的局部治疗手段，其并发症的发生情况，依据美国介入放射学会（Society of Interventional Radiology, SIR）的标准^[177]^进行评估分级。按照发生时间分为即刻并发症（消融后 < 24 h）、围手术期并发症（消融后24 h-30 d）及迟发并发症（消融后 > 30 d）（表 1）。

**表 1:** 美国介入放射学会对并发症的定义与分级标准 Society of Interventional Radiology standard for definition and grading of complications

并发症分类	定义
副反应	①疼痛； ②消融后综合症； ③无症状胸腔积液； ④无后果的邻近结构损伤。
轻微	①无不良结果，不需要治疗； ②无不良结果，仅需要对症治疗或过夜观察。
严重	①需要住院治疗或住院时间延长≤48 h； ②需要住院进行较大治疗，提升护理级别； ③延长住院时间 > 48 h； ④导致永久不良后遗症； ⑤死亡。

### 12.1 不良反应

#### 12.1.1 疼痛

在局麻条件下消融，一般均有不同程度的疼痛（尤其是邻近胸膜的疾病）。如果疼痛剧烈，可以加大阿片类止痛药物的用量，同时可以给予适量镇静剂。手术后疼痛一般为轻度疼痛，可持续数天，也有人持续1周-2周，很少出现中度以上的疼痛，可以用非甾体类药物止痛。

#### 12.1.2 消融后综合征

约1/3的患者可能发生，是由于坏死物质的吸收和炎性因子的释放引起。主要症状为低热、乏力、全身不适、恶心、呕吐等，一般持续3 d-5 d。这种情况对症处理即可，必要时除给予非甾体类药物外，可以适量短时应用小剂量糖皮质激素。

#### 12.1.3 咳嗽

消融术中出现咳嗽是十分常见的症状，剧烈的咳嗽可导致或加重气胸或皮下气肿，有时可使消融针移位。轻度的咳嗽不影响消融手术，剧烈咳嗽要停止消融手术或间断消融。引起咳嗽的原因可能与消融时局部温度增高刺激肺泡、支气管内膜或胸膜所致，术后咳嗽是肺结节组织坏死及其周围肺组织热损伤引起的炎症反应所致。术前1 h口服可待因可减轻咳嗽反应。术后咳嗽可适当给予止咳化痰药以及必要的抗生素。

#### 12.1.4 胸膜反应

消融过程中刺激了支配壁层胸膜的迷走神经，兴奋的迷走神经可使心率减慢，甚至心跳停止。出现这种情况需暂停消融，要充分局部麻醉，并适当应用阿托品、镇静剂等药物。

### 12.2 并发症

#### 12.2.1 气胸

气胸是消融后最常见的并发症，发生率为50%左右。气胸更常见于以下情况：肺气肿、男性、年龄 > 60岁、结节位于肺下叶、单发结节穿刺肺组织次数 > 3次、多发肺GGN消融多个结节穿刺次数多、穿过叶间裂、消融与活检同步或序贯进行。大部分气胸容易治疗，或者是自限性的，不需要治疗即可自愈，需要胸腔闭式引流的15%。如果患者经过胸腔闭式引流仍然有气体漏出，可以持续负压吸引、行胸膜固定术、气管镜下注入硬化剂、气管内置入阀门、胸腔镜手术修补等。另外，要注意迟发性气胸的发生。

#### 12.2.2 胸腔积液

消融后经常可以见到少量胸腔积液，发生率为30%，被认为是机体对热损伤的交感反应，需要穿刺或置管引流的胸腔积液占5%。导致胸腔积液发生的危险因素包括大病灶、一次消融多个病灶、病灶靠近胸膜、消融时间长等。

#### 12.2.3 出血

消融中出血的发生率为3%-8%，出血表现为咯血、血胸、失血性休克和急性呼吸衰竭，但主要表现为咯血和血胸。①咯血：出现中等以上的咯血时应立即消融病灶，同时静脉输注止血药。由于消融本身可以使血液凝固，随着消融治疗的进行出血会逐渐停止，故在具体消融治疗过程中大出血的发生率并不高。术后咯血多具有自限性，可持续3 d-5 d。保守治疗无效者，可行介入栓塞治疗或剖胸探查；②血胸：主要是因为在穿刺过程中损伤了胸廓内动脉、肋间动脉或其他动脉等。在穿刺过程中要避免穿刺到上述动脉，如果出现血胸要密切观察、积极治疗，保守治疗无效者可行介入栓塞治疗或剖胸探查。

#### 12.2.4 感染

对于老年并伴有重度基础肺部疾病的患者，肺部感染的机会更多，术前30 min-1 h可以预防性应用抗生素，24 h内再用一次。在下列情况下消融手术后预防性应用抗生素可以适当延长到48 h-72 h：老年人 > 70岁、长期慢性阻塞性肺气肿、糖尿病控制欠佳、单侧肺GGN消融数量 > 3个、免疫力低下等。若消融手术后5 d体温仍然 > 38.5 ℃，首先要考虑肺部感染，要根据痰液、血液或脓液培养的结果调整抗生素。如果发生肺部或胸腔脓肿可以置管引流并冲洗。

#### 12.2.5 空洞形成

空洞形成是肺部肿瘤热消融后的常见征象，可以视为术后的自然转归过程，但是也可能成为感染、出血等严重并发症的根源。大多术后15 d-1个月出现，2个月-4个月后吸收。大部分空洞没有症状，不需处理。如果出现发热、咳浓痰，应考虑空洞感染、脓肿形成，脓肿要及时引流。另外，要警惕曲霉菌感染。

#### 12.2.6 其他

少见并发症：支气管胸膜瘘、非靶区热灼伤或冻伤、肋骨骨折、冷休克、血小板降低、神经损伤（臂丛、肋间、膈、喉返神经等）、肺栓塞、空气栓塞、心包填塞等需个别特殊处理。

## 13 结语

随着社会经济的发展、科学技术的进步和国民收入的增加，人们对健康保健需求提出了更高的标准，不但要有强壮的身体，还要有良好的心理状态和社会活动能力。根据人们对健康保健的新需求，传统的以诊疗“疾病”为中心的“生物医学”模式势必要转变为以“人”为中心的“生物-心理-社会”医学模式。LDCT筛查和由MDT讨论形成的对GGN处理意见作为典型的“生物医学”产物，一方面在发现早期肺癌和降低肺癌死亡率方面发挥了积极作用，另一方面也给人们带来了一系列的心理、社会和经济问题。因此要从“生物-心理-社会”多维度去正确认识LDCT筛查和MDT讨论意见，要把SDM融合到GGN的整个诊疗过程中，使患者获益最大化且风险最小化。另外，LDCT只是肺癌筛查的一项影像学技术，生物标志物与影像学联合筛查模式，可能更有助于肺癌的早期诊断，因此寻找敏感性高和特异性高的生物标志物是今后癌症筛查的方向之一。AI和云端技术是推动大数据创新应用的重要手段，也是医疗卫生健康领域未来发展趋势^[178]^。AI+云端技术形成的“物联网医学”^[179]^将有助于GGN同质化管理、多学科专家远程会诊和随访。

目前肺部GGN仍以局部治疗为主，热消融做为局部微创治疗技术之一，虽然在治疗GGN（尤其对于多发GGN）方面有一定优势，但是还存在许多问题：①缺乏大规模的、多中心的、前瞻性的临床研究；②缺乏长期（10年以上）临床疗效的随访结果；③缺乏与其他传统治疗手段（如VATS）的前瞻性的、随机的、多中心的临床比较研究；④如何精准定位，提高活检阳性率和局部完全消融率，是今后工作的方向之一；⑤电磁导航下肺GGN热消融技术也在发展，同时也显示了一定优势，但是普及该技术可能比较困难；⑥作为我国的“限制性医疗技术”，由于治疗设备的生产厂家不同，设备性能之间的差异，再加上该专业刚刚兴起，治疗人员的专业化水平参差不齐，现在很难形成公认的治疗规范和标准；⑦基础研究相对滞后，如复杂热场分布等；⑧人们对热消融技术治疗GGN还存在一定疑问，需要进一步开展工作以改变传统思维对热消融技术的认知，使得该技术得以普及和应用。
